# Clinical Outcomes of Micafungin and Anidulafungin in *Candidozyma auris* (Formerly *Candida auris*) Candidemia: A Propensity Score-Matched Retrospective Cohort Study

**DOI:** 10.3390/jof12070537

**Published:** 2026-07-22

**Authors:** Eyüp Arslan, Ömer Karaşahin, Betül Yüsra Şirin, Deniz Turan, Yıldız Olçar, Umut Elmas, Fatma Yılmaz Karadağ, Derya Öztürk Engin

**Affiliations:** 1 Sancaktepe Şehit Prof. Dr. İlhan Varank Training and Research Hospital, Department of Infectious Diseases and Clinical Microbiology, University of Health Sciences, Istanbul 34785, Türkiye; 2 Erzurum Training and Research Hospital, Department of Infectious Diseases and Clinical Microbiology, University of Health Sciences, Erzurum 25040, Türkiye; 3 Sancaktepe Şehit Prof. Dr. İlhan Varank Training and Research Hospital, Department of Anesthesiology and Reanimation, University of Health Sciences, Istanbul 34785, Türkiye; 4 Haydarpaşa Numune Training and Research Hospital, Laboratory of Medical Microbiology, University of Health Sciences, Istanbul 34668, Türkiye

**Keywords:** *Candida auris*, *Candidozyma auris*, candidemia, micafungin, anidulafungin, echinocandins, propensity score matching, drug-induced liver injury

## Abstract

*Candidozyma auris* (formerly *Candida auris*) is a critical-priority multidrug-resistant pathogen. Comparative clinical data on first-line echinocandins—micafungin and anidulafungin—in *C. auris* candidemia remain limited. This retrospective cohort study compared clinical outcomes of micafungin and anidulafungin in adult patients with *C. auris* candidemia treated between January 2024 and December 2025 at three affiliated hospital campuses in Istanbul, Türkiye. Propensity score matching (PSM) using a 1:1 nearest-neighbor algorithm was performed to balance baseline characteristics. Outcomes included 30-day (primary) and 14-day all-cause mortality, microbiological response, end-of-therapy (EOT) response, relapse, and drug-induced liver injury assessed by the Roussel Uclaf Causality Assessment Method (RUCAM). Among 154 included patients (micafungin, n = 94; anidulafungin, n = 60), no echinocandin resistance was detected. After PSM (55 matched pairs), 30-day all-cause mortality was identical between groups (41.8% vs. 41.8%; mOR 1.00, 95% CI 0.43–2.31; *p* = 1.000). Fourteen-day all-cause mortality (16.4% vs. 18.2%; *p* = 0.763), microbiological response (94.5% vs. 90.9%; *p* = 0.480), EOT response (74.5% vs. 67.3%; *p* = 0.346), and relapse (12.7% vs. 10.9%; *p* = 0.763) did not differ significantly between groups. RUCAM-based hepatic safety profiles were descriptively comparable. Micafungin and anidulafungin showed comparable observed outcomes in *C. auris* candidemia in this cohort.

## 1. Introduction

*Candidozyma auris* (formerly *Candida auris*) is a multidrug-resistant yeast first described in 2009 [1]. Since then, it has spread rapidly worldwide and caused outbreaks in hospital settings [2]. The World Health Organization classified it as a critical priority pathogen in 2022 [3]. *C. auris* is among the most difficult pathogens to control in healthcare facilities. It survives for prolonged periods on medical devices and hospital surfaces, forms biofilms, and resists standard disinfection protocols [4]. Antifungal resistance is another defining feature of *C. auris*, although its extent varies by clade and geographic region. Fluconazole resistance is highly prevalent, exceeding 90% among isolates from regions where Clade I and Clade III predominate, whereas Clade IV isolates are comparatively more susceptible (~11% resistance) [5]. Moreover, some strains are resistant to all available antifungal classes [6]. Consistent with this resistance profile, 30-day mortality in *C. auris* candidemia is high, ranging from 23% to 67% [3].

The most recent global guideline recommends echinocandins (anidulafungin, caspofungin, micafungin, and rezafungin) as first-line agents for candidemia and invasive candidiasis, and considers their spectra of activity equivalent. However, no evidence-based guidance is available on which echinocandin should be preferred in *C. auris* candidemia [7]. Micafungin and anidulafungin are the echinocandins most frequently used for *C. auris* candidemia within our hospital network because of their availability [8]. Yet comparative data on clinical or microbiological superiority between these two agents remain limited. Available studies are largely based on small samples or on retrospective cohorts that do not control for baseline imbalances [9,10]. In a retrospective cohort from India, no significant differences were found among three echinocandins in clinical and microbiological outcomes; however, that study did not apply any method to balance baseline differences between groups [11]. Hepatic safety is another factor that influences echinocandin selection. Micafungin undergoes enzymatic metabolism in the liver, whereas anidulafungin is eliminated through slow spontaneous biotransformation [12]. This difference has raised clinical concerns about the hepatotoxicity risk of micafungin.

In this retrospective cohort study, we aimed to compare the effectiveness and safety of micafungin and anidulafungin in patients with *C. auris* candidemia using propensity score matching (PSM), which, to our knowledge, has not previously been applied to this comparison. The primary objective was to compare 30-day all-cause mortality between PSM treatment groups. Secondary objectives included comparisons of 14-day all-cause mortality, microbiological response, end-of-therapy response, relapse, and drug-induced liver injury (DILI), assessed using the Roussel Uclaf Causality Assessment Method (RUCAM). The study also aimed to descriptively characterize the entire cohort before PSM.

## 2. Materials and Methods

This was a retrospective cohort study conducted at a single tertiary care center comprising three affiliated hospital campuses (Sancaktepe Şehit Prof. Dr. İlhan Varank Training and Research Hospital, Prof. Dr. Feriha Öz Emergency Hospital, and Çekmeköy State Hospital) located in Istanbul, Türkiye. The three campuses operate under a single administrative management, with infectious diseases consultation services provided by a unified clinical team. Accordingly, this cohort is best regarded as a functionally single-center study. The study period extended from 1 January 2024 to 31 December 2025.

### 2.1. Study Population

Adult patients with *C. auris* candidemia were identified during the study period. Case identification was performed through records maintained by the infection control unit, and clinical and laboratory data were retrospectively retrieved from the hospital information system.

Inclusion criteria were: (i) age ≥18 years; (ii) at least one blood culture positive for *C. auris*; (iii) use of either micafungin or anidulafungin as primary antifungal therapy; and (iv) completion of a minimum of 72 h of the initial antifungal treatment. Exclusion criteria were: (i) initial therapy with antifungal agents other than micafungin or anidulafungin, (ii) switching between echinocandins during therapy, or use of combination antifungal therapy; (iii) mixed candidemia with concurrent isolation of additional *Candida* species.

In patients with recurrent candidemia episodes, only the first episode was included in the analysis. The choice between micafungin and anidulafungin was not protocol-driven. In routine practice, it was guided mainly by drug availability at the time of treatment, physician preference, and administration practicality, as micafungin is administered as 100 mg once daily without a loading dose, whereas anidulafungin requires a 200 mg loading dose followed by 100 mg once daily [12].

### 2.2. Definitions

Microbiological response: To monitor microbiological eradication, blood cultures were obtained every 48 h in patients diagnosed with *C. auris* candidemia who had initiated antifungal therapy. Microbiological response was defined as three consecutive negative blood cultures, obtained at 48 h intervals, with no growth of *C. auris*.

End-of-therapy (EOT) response: EOT response was defined as defervescence, documented clearance of *C. auris* from blood cultures, and completion of at least 14 days of antifungal therapy from the date of the first negative blood culture of the microbiological response.

Relapse: In-hospital relapse was defined as a new episode of *C. auris* candidemia occurring after discontinuation of antifungal therapy in patients who had previously achieved an EOT response, monitored until death or hospital discharge, whichever occurred first.

Drug-induced liver injury (DILI): Hepatotoxicity following antifungal initiation was defined according to Danan and Teschke as alanine aminotransferase (ALT) ≥5× upper limit of normal (ULN) and/or alkaline phosphatase (ALP) ≥2× ULN. In cases meeting these biochemical criteria, the pattern of injury was classified as hepatocellular, cholestatic, or mixed using the R ratio (ALT/ULN ÷ ALP/ULN), and causality was assessed with the updated RUCAM scale appropriate to the injury pattern. Total RUCAM scores were graded as: excluded (≤0), unlikely (1–2), possible (3–5), probable (6–8), and highly probable (≥9) [13].

### 2.3. Microbiological Identification and Susceptibility Testing

Blood culture isolates with yeast growth were referred to the central mycology reference laboratory (Istanbul Public Health Laboratory-2) for species identification and antifungal susceptibility testing. Species identification was performed by MALDI-TOF MS [VITEK^®^ MS (bioMérieux, Marcy l’Etoile, France)]. Antifungal susceptibility testing was performed using the Sensititre YeastOne (SYO) colorimetric microdilution panel (Thermo Fisher Scientific, Waltham, MA, USA) for anidulafungin, caspofungin, micafungin, amphotericin B, and fluconazole; minimum inhibitory concentration (MIC) values were interpreted according to the tentative breakpoints recommended by the Centers for Disease Control and Prevention (CDC) for *C. auris* [14].

### 2.4. Data Collection

Data were retrospectively retrieved from the hospital electronic information system using a standardized form. Collected variables included demographic characteristics at hospital admission, medical history, comorbidities, and the Charlson Comorbidity Index (CCI) [15]; total length of hospital stay before candidemia, invasive procedures and medical support therapies; prior *C. auris* colonization and concurrent bacteremia; antibiotic regimens used during the 30 days preceding candidemia and on the day of candidemia; and laboratory parameters, vasopressor use, and Sequential Organ Failure Assessment (SOFA) score on the day of candidemia [16]. Antifungal-related variables included time from the first positive blood culture to antifungal initiation, type and total duration of antifungal therapy, microbiological response, EOT response, relapse, 14- and 30-day all-cause mortality, and length of hospital stay after candidemia. Adherence to clinical guidelines in antifungal management was assessed using the EQUAL *Candida* Score [17]. For patients meeting DILI biochemical criteria, RUCAM domains—time to onset from antifungal initiation, course of liver enzymes after dechallenge, risk factors, concomitant hepatotoxic medications, alternative causes (viral hepatitis serology, hepatobiliary imaging, hemodynamic and septic complications), prior reports of antifungal hepatotoxicity, and any unintentional reexposure—were scored from hospital records. Where data required for a given RUCAM domain were unavailable, the item was scored conservatively as 0 (‘no information’).

### 2.5. Statistical Analysis

To minimize treatment selection bias inherent to the retrospective design, PSM was performed between patients with *C. auris* candidemia receiving micafungin and those receiving anidulafungin. Only variables available at or before the initiation of antifungal therapy were included in the propensity model. The following covariates were entered into the propensity model: demographics (age, sex), comorbidity burden (Charlson Comorbidity Index), disease severity (SOFA score), intensive care unit (ICU) admission at candidemia onset, invasive support (mechanical ventilation, vasopressor use, total parenteral nutrition), abdominal surgery within the preceding 3 months, concurrent bacteremia, antifungal delay (time from index culture to antifungal initiation), pre-candidemia hospital length of stay, prior *C. auris* colonization, and C-reactive protein level at candidemia onset. Propensity scores were calculated using a logistic regression model. Patients were matched 1:1 using nearest-neighbor matching without replacement, with a caliper width of 0.2 standard deviations of the logit of the propensity score. No covariate included in the propensity-score model had missing values; imputation was therefore not required, and all eligible patients were included in the propensity-score estimation. Patients for whom no suitable match was found within the caliper were excluded from the matched cohort. Covariate balance was assessed using the absolute standardized mean difference (SMD); an absolute SMD <0.10 was considered indicative of adequate balance.

Categorical variables are presented as numbers (percentages) and continuous variables as medians (interquartile range [IQR]). For descriptive between-group comparisons before and after matching, categorical variables were compared using the chi-square test or Fisher’s exact test, and continuous variables were compared using the Mann–Whitney U test. Covariate balance after propensity score matching was primarily assessed using absolute standardized mean differences rather than *p*-values. The unmatched cohort was used for descriptive comparisons, whereas treatment comparisons for the study outcomes were performed in the matched cohort. In the matched cohort, the primary outcome (30-day all-cause mortality) and binary secondary clinical outcomes (14-day all-cause mortality, microbiological response, end-of-therapy response, and relapse) were analyzed according to the matched-pair structure. Matched odds ratios (mORs) with 95% confidence intervals were calculated from discordant matched pairs, and *p*-values were obtained using the asymptotic McNemar test. RUCAM categories were summarized descriptively because of sparse cell counts; category-specific odds ratios and *p*-values were not estimated.

For survival analyses, time zero was defined as the date of the first positive blood culture indicative of *C. auris* candidemia onset. The event of interest was all-cause mortality. Fourteen-day and 30-day survival analyses were conducted separately; patients who died within the respective time window were considered events, while survivors were censored at day 14 or day 30, respectively. Survival probabilities for the micafungin and anidulafungin groups were estimated using the Kaplan–Meier method and compared using the log-rank test. All Kaplan–Meier analyses were performed in the propensity score-matched cohort. A two-sided *p*-value < 0.05 was considered statistically significant. All statistical analyses were performed using IBM SPSS Statistics for Windows, version 31.0.2.0 (IBM Corp., Armonk, NY, USA).

### 2.6. Ethics Statement

This study was approved by the Scientific Research Ethics Committee of the University of Health Sciences, Sancaktepe Şehit Prof. Dr. İlhan Varank Training and Research Hospital (Approval date: 04 February 2026; Decision No: 54). The requirement for informed consent was waived by the Ethics Committee due to the retrospective design of the study. The study was conducted in accordance with the principles of the Declaration of Helsinki.

## 3. Results

### 3.1. Study Population

**Figure 1:**
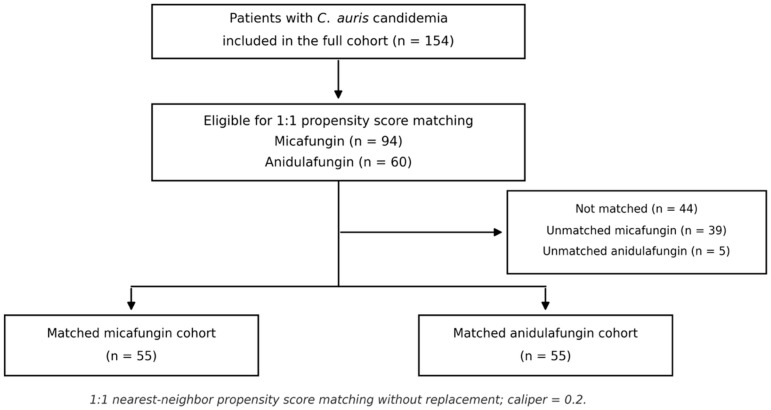
Study flow diagram of patient selection and propensity score matching.

A total of 154 adult patients with *C. auris* candidemia were included in the cohort. Of these, 94 (61.0%) received micafungin, and 60 (39.0%) received anidulafungin. After PSM, 55 matched pairs (110 patients) were obtained; 39 (41.5%) micafungin patients and 5 (8.3%) anidulafungin patients were excluded because no suitable match was found. The study flow diagram is shown in Figure 1.

Covariate balance before and after PSM was evaluated using the absolute standardized mean differences (SMD) presented in Supplementary Table S1, together with the graphical representation in Supplementary Figure S1. Before PSM, limited imbalances were observed among the selected covariates, with more pronounced differences in total parenteral nutrition use (SMD = 0.230), abdominal surgery within the preceding 3 months (SMD = 0.244), and prior *C. auris* colonization (SMD = 0.230). After PSM, all covariates achieved acceptable balance, with absolute standardized mean differences below 0.1, as detailed in Supplementary Table S1.

### 3.2. Baseline Characteristics

The baseline characteristics of the micafungin and anidulafungin groups before and after PSM are presented in Table 1. Before PSM, the two groups were broadly similar with respect to demographic characteristics, comorbidities, and most key clinical variables. After matching, a balanced distribution of baseline characteristics was achieved between the groups. In the matched cohort, the proportion of female patients was 45.5% in both groups; the median age was 70 years (IQR, 63.5–81.5) in the micafungin group and 70 years (IQR, 61.5–80.5) in the anidulafungin group. Similarly, no statistically significant differences were found between the groups with respect to BMI, ICU admission on the day of culture, CCI, SOFA score, antifungal use within the preceding month, antifungal resistance rates, concurrent bacteremia, prior *C. auris* colonization, length of hospital stay before candidemia, time from culture to antifungal initiation, total duration of antifungal therapy, and length of hospital stay after candidemia (all *p* > 0.05).

### 3.3. Antifungal Susceptibility

Among the 154 *C. auris* isolates in the overall cohort, amphotericin B resistance was detected in 125 isolates (81.2%) and fluconazole resistance in 147 isolates (95.5%). No isolates resistant to the three tested echinocandins (anidulafungin, micafungin, and caspofungin) were observed. Antifungal resistance rates were similarly distributed between the micafungin and anidulafungin groups (Table 1; all *p* > 0.05).

### 3.4. Invasive Devices and Supportive Therapies

The use of invasive devices and supportive therapies in the micafungin and anidulafungin groups before and after PSM is presented in Table 2. No statistically significant differences were found between the groups in the rates of central venous catheter, mechanical ventilation, tracheostomy, surgical drain catheter, vasopressor use, or total parenteral nutrition, either before or after matching (all *p* > 0.05). Urinary catheter use was high in both groups and remained similar after matching. Central venous catheter removal rates also did not differ significantly between the matched groups. The timing of central venous catheter removal—categorized according to the EQUAL *Candida* score as within 24 h, between 24 and 72 h, and after 72 h of diagnosis, together with catheters not removed—was also similar between the groups before and after matching (*p* = 0.263 and *p* = 0.703, respectively; Supplementary Table S2). No significant differences were found between the groups for less frequent variables such as history of abdominal surgery within the preceding 3 months and need for hemodialysis.

### 3.5. Laboratory Parameters and Antibiotic Use

Laboratory parameters of the micafungin and anidulafungin groups before and after PSM are presented in Table 3. No statistically significant differences were observed between the groups in white blood cell (WBC), neutrophil, and platelet counts, or in procalcitonin, C-reactive protein (CRP), and ALT levels, either before or after matching (all *p* > 0.05). Concurrent antibiotic use on the day of culture is summarized in Supplementary Table S3. Before PSM, no statistically significant differences were found between the micafungin and anidulafungin groups in the rates of carbapenem, glycopeptide, colistin/polymyxin, piperacillin–tazobactam, quinolone, macrolide, and third-generation cephalosporin use (all *p* > 0.05). After matching, the distribution of antibiotic use remained similarly balanced between the two groups. In the matched cohort, no significant differences were observed in the rates of carbapenem, glycopeptide, colistin/polymyxin, and piperacillin–tazobactam use (all *p* > 0.05). Among the less frequently used antibiotics—including quinolones, macrolides, third-generation cephalosporins, ceftazidime–avibactam, ampicillin–sulbactam, aminoglycosides, and fosfomycin—the differences between groups did not reach statistical significance (Supplementary Table S3).

### 3.6. Primary and Secondary Outcomes

In the overall cohort (n = 154), 30-day all-cause mortality occurred in 67 patients (43.5%), 14-day mortality in 30 patients (19.5%), microbiological response in 143 patients (92.9%), EOT response in 110 patients (71.4%), and relapse in 18 patients (11.7%). Primary and secondary outcomes in the propensity score-matched cohort are presented in Table 4. In the matched cohort, 30-day all-cause mortality was 41.8% in both groups (*p* = 1.000). Similarly, 14-day all-cause mortality was 16.4% in the micafungin group and 18.2% in the anidulafungin group, with no significant difference between groups (*p* = 0.763). Microbiological response was higher in the micafungin group but did not reach statistical significance (94.5% vs. 90.9%; *p* = 0.480). EOT response rates were also similar between the two groups (74.5% vs. 67.3%; *p* = 0.346). No significant difference was found in relapse frequency (12.7% vs. 10.9%; *p* = 0.763). In the overall cohort, hepatic enzyme elevation was detected in 17 patients (11.0%) and assessed using RUCAM. The distribution by RUCAM category was unlikely (n = 8), possible (n = 8), and probable (n = 1); no patient had DILI at the highly probable level, and the single probable case occurred in the micafungin group. After PSM, the RUCAM distribution in the matched cohort was limited to the unlikely and possible categories and was descriptively similar between groups (Table 4). Because of sparse category counts, category-specific inferential comparisons were not performed.

### 3.7. Survival Analysis

**Figure 2:**
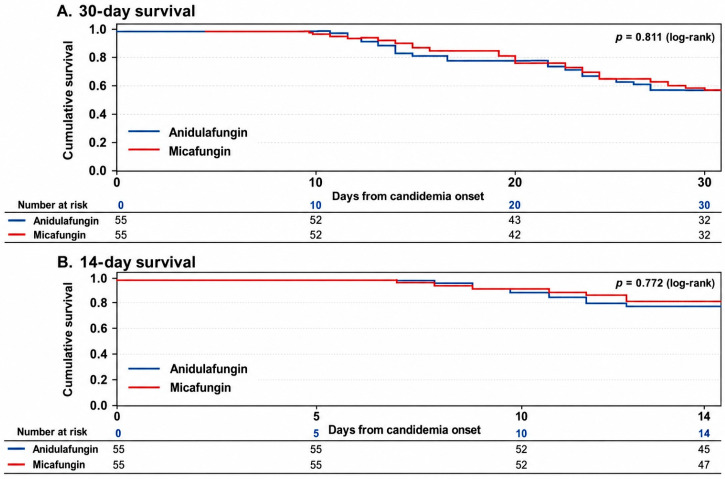
Kaplan–Meier survival estimates by echinocandin treatment in the propensity score-matched cohort: 30-day survival (**A**) and 14-day survival (**B**).

In the propensity score-matched cohort, 30-day and 14-day survival by echinocandin treatment were evaluated using the Kaplan–Meier method (Figure 2). The log-rank test showed no statistically significant difference between the micafungin and anidulafungin groups for 30-day survival (*p* = 0.811) or 14-day survival (*p* = 0.772). These findings indicate no significant difference in short-term survival between the two groups in the matched cohort.

## 4. Discussion

In this retrospective cohort study, we compared the clinical outcomes of micafungin and anidulafungin in patients with *C. auris* candidemia using PSM to balance baseline characteristics. The primary endpoint of 30-day all-cause mortality was similar between the micafungin and anidulafungin groups. Secondary clinical endpoints, including 14-day all-cause mortality, microbiological response, end-of-therapy response, and relapse, did not differ significantly between the two groups. RUCAM-based hepatic safety profiles were descriptively comparable between groups.

Our findings indicate that the similar in vitro activity of micafungin and anidulafungin was accompanied by comparable observed clinical outcomes in this cohort. Analyses by the European Committee on Antimicrobial Susceptibility Testing (EUCAST) have shown that micafungin and anidulafungin have identical MIC50 (0.06 mg/L) and epidemiological cutoff (ECOFF) values (0.25 mg/L) against *C. auris* [18]. At the clinical level, a retrospective cohort from India found no significant differences among three echinocandins (micafungin, anidulafungin, and caspofungin) in clinical and microbiological outcomes [11]. These observations are also consistent with the current global guideline, which recommends echinocandins as first-line agents for the treatment of candidemia and invasive candidiasis [7]. Our findings support that micafungin and anidulafungin may be clinically comparable even when the influence of confounding clinical factors is reduced through PSM.

In our propensity score-matched cohort, no significant difference in 30-day or 14-day all-cause mortality was found between the micafungin and anidulafungin groups, and the Kaplan–Meier survival curves did not diverge. These findings support that the short-term clinical efficacy of the two echinocandins in *C. auris* candidemia may be comparable. The 30-day mortality rate in our overall cohort (43.5%) falls within the 23–67% range reported in the systematic review by Kim et al. [3]. However, it is relatively higher than the 38.1% reported in the multicenter Colombian cohort of Ortiz-Roa et al. and the 37% reported by Pandya et al. [19,20]. As noted by Kim et al., *C. auris*-related mortality is multifactorial, driven primarily by comorbidities, complications of invasive procedures, and delays in diagnosis and treatment [3]; the high CCI scores and the proportion of patients managed in the intensive care setting in our cohort reflect this multiple-risk profile. Furthermore, in the study by Pandya et al., *C. auris* was isolated from blood in 76% of cases, whereas all patients in our study had candidemia, which limits direct comparison between the cohorts [20]. Mortality in this study was assessed as all-cause rather than *C. auris*-attributable. In this critically ill, highly comorbid population, mortality is multifactorial, and reliable attribution of individual deaths to *C. auris* was not feasible in this retrospective cohort. All-cause mortality, therefore, provides a reproducible outcome that does not depend on cause-of-death adjudication. This was a pre-specified choice, consistent with most comparable cohorts, in which crude (all-cause) mortality predominates and attributable mortality is infrequently reported and difficult to ascertain [3].

In both groups compared in the current study, no significant differences were found in the other secondary outcomes—microbiological response, EOT response, and relapse rates. These findings indicate that micafungin and anidulafungin have similar characteristics with respect to the secondary outcomes assessed. This result is consistent with Prayag et al., who found no differences in clinical and microbiological responses among micafungin, anidulafungin, and caspofungin in *C. auris* candidemia when a susceptible echinocandin was selected [11]. Studies directly comparing micafungin and anidulafungin in non-*C. auris* candidemia have reported similar findings. In a retrospective cohort of 98 adult patients with candidemia conducted in Korea by Suh et al., clinical response (51.9% vs. 46.7%) and microbiological response (76.9% vs. 67.4%) rates were similar between the two groups [21]. In the study by van der Geest et al., which included 63 patients with invasive candidiasis in the intensive care unit, global response (67% vs. 70%), clinical response (80% vs. 85%), and microbiological response (70% vs. 73%) rates did not differ significantly between the micafungin and anidulafungin groups [22]. Using a similar propensity score-matched design, Elajez et al. recently compared caspofungin and anidulafungin in patients with invasive candidiasis and, consistent with our findings, observed no significant differences in global, clinical, or mycological response [23]. These findings indicate that comparable outcomes among different echinocandins have been observed consistently across studies. As the present study compared only micafungin and anidulafungin, its findings cannot be extrapolated to other echinocandins.

In the overall cohort, a relapse rate of 11.7% was observed after echinocandin therapy concluded with an EOT response. This finding is consistent with the pattern of increased recurrence risk in *C. auris* candidemia compared with other *Candida* spp. In the multicenter study by Simon et al., the microbiological recurrence rate within 60 days after the last dose of antifungal therapy was reported as 11.9% in *C. auris* candidemia; in the propensity score-adjusted analysis, *C. auris* was identified as an independent risk factor for recurrence compared with other *Candida* species (aOR 4.46, 95% CI 1.03–19.26; *p* = 0.04) [24]. In our study, relapse rates did not differ significantly between micafungin and anidulafungin treatments. However, the main factor influencing relapse may be patient-related factors rather than the type of echinocandin used. *C. auris* can persistently colonize the skin of patients even after successful treatment [25]. This persistent colonization, together with other established risk factors for candidemia, may create a predisposition for recurrence [26].

In the overall cohort, hepatic enzyme elevations meeting DILI biochemical criteria (ALT ≥5× ULN or ALP ≥2× ULN) were observed in 17 patients, and RUCAM assessment placed most cases in the unlikely and possible categories. One patient treated with micafungin was classified as having DILI in the probable category. After PSM, the RUCAM distribution remained descriptively similar between the two groups. This finding indicates that the hepatic safety profiles of micafungin and anidulafungin may be similar. Our results are consistent with the hepatic safety findings of studies directly comparing the two agents. Suh et al. assessed hepatotoxicity in micafungin and anidulafungin groups in patients with candidemia using the Common Terminology Criteria for Adverse Events (CTCAE) version 5 and found no significant difference between the two groups [21]. In the study by van der Geest et al., no significant differences were observed between the two agents in ALT, aspartate aminotransferase, prothrombin time, and bilirubin levels [22]. However, in these studies, DILI was not assessed with a standardized causality tool; hepatotoxicity was defined solely on the basis of enzyme elevation or CTCAE grading. Yet the choice of causality assessment method substantially affects the estimated frequency of DILI; when Mullins et al. assessed micafungin-associated DILI using RUCAM and Naranjo, they recommended RUCAM as the more sensitive tool [27]. Because anidulafungin does not undergo hepatic metabolism, it is frequently preferred in patients with hepatic dysfunction, which may lead to indication bias in observational studies [28]. In our overall cohort, the median (IQR) baseline ALT was 20 (11–35), and only 2 patients (1.3%) had chronic liver disease, which minimizes this bias. Under conditions in which confounding was reduced through PSM, no difference in RUCAM-based hepatic safety was observed between the two agents.

Regarding the antifungal resistance profile in our study, the amphotericin B resistance rate (81.2%) was notably high. In the United States, the amphotericin B resistance rate among *C. auris* clinical isolates tested by the Antimicrobial Resistance Laboratory Network during 2022–2023 was reported as 15% [29]. This discrepancy may be attributable to the tendency of the SYO method used in our laboratories to overestimate amphotericin B MIC values in *C. auris* isolates. Siopi et al. showed that MIC values obtained with SYO were 1–2 dilutions higher than those obtained with the CLSI reference method, resulting in 89% major errors when the CDC breakpoint (≥ 2 mg/L) was applied [30]. This methodological limitation should be taken into account when interpreting our amphotericin B susceptibility data. With respect to azole susceptibility, fluconazole resistance in our cohort was high (95.5%). Fluconazole resistance in *C. auris* is clade- and region-dependent, being particularly high among Clade I and Clade III isolates [5]. A whole-genome sequencing-based phylogenetic analysis from Türkiye has identified Clade I (South Asian) in our region [31]. The high fluconazole resistance observed in our cohort is consistent with the high azole resistance characteristic of this clade, and with earlier Türkiye isolates in which fluconazole MICs were uniformly high [32]. No echinocandin resistance (to anidulafungin, micafungin, or caspofungin) was detected in our cohort. Although clade typing was not performed on our isolates, echinocandin resistance in *C. auris* has remained low both regionally—including in Clade I isolates reported from Türkiye, which were susceptible to anidulafungin and micafungin [31]—and globally (<5% across clades), despite sporadic and emerging reports [5]. These resistance data form part of the pre-specified descriptive analysis of the whole cohort and define the multidrug-resistant context in which echinocandins are used first-line, motivating the present comparison.

To our knowledge, this is the first and largest cohort study to compare micafungin and anidulafungin in propensity score-matched groups in *C. auris* candidemia. The strengths of the study include a homogeneous patient group consisting solely of candidemia cases, the management of all patients by a single clinical team with a standardized approach, and the assessment of hepatic safety using RUCAM, a hepatotoxicity-specific causality tool that provides more structured assessment than enzyme-elevation or CTCAE-based criteria [21,22]. A further strength is the use of PSM, which achieved balance across all measured baseline covariates (absolute standardized mean differences <0.10), including covariates that were imbalanced before matching; by reducing confounding by indication, this strengthens the internal validity of the treatment comparison relative to unadjusted analyses. Nevertheless, some limitations should be considered. The retrospective design of the study cannot fully exclude the effect of unmeasured confounding factors that could not be balanced by PSM. However, the choice between agents was driven mainly by factors independent of patient prognosis—drug availability, physician preference, and administration practicality—and the two groups were balanced on comorbidity and severity indices even before matching, which limits the likelihood of substantial indication bias. In addition, RUCAM was applied retrospectively rather than prospectively as originally recommended by Danan and Teschke [13]; domains with incomplete data were scored conservatively, potentially biasing causality scores toward lower categories. Moreover, DILI events were infrequent, and cases in the higher RUCAM categories were rare; the hepatic safety comparison should therefore be interpreted as descriptive and hypothesis-generating rather than as evidence of equivalent hepatotoxicity. Separately, blood cultures used to determine microbiological eradication were obtained every 48 h rather than at the daily interval recommended by current guidelines, owing to the high case volume and laboratory capacity constraints within our hospital network. This may have affected the precision of the total treatment duration after microbiological eradication. The single-center nature of the cohort may limit the generalizability of the findings to different *C. auris* clades and geographic regions. In this regard, molecular clade typing was not performed on our isolates, and systematic multicenter clade surveillance data from Türkiye remain scarce, precluding clade-specific analyses. Finally, the matched sample size (55 pairs) was modest, and the confidence intervals for several outcomes were wide; the absence of statistically significant differences may reflect limited statistical power (type II error) and does not exclude smaller but potentially clinically meaningful differences between the agents. These limitations indicate that the comparable outcomes observed in this cohort should be confirmed by prospective, multicenter studies.

## 5. Conclusions

In this propensity score-matched cohort, micafungin and anidulafungin showed comparable outcomes in *C. auris* candidemia with respect to mortality, microbiological response, end-of-therapy response, and relapse, with descriptively comparable RUCAM-based hepatic safety. As the study was not designed to establish equivalence or non-inferiority, these findings indicate comparable observed outcomes rather than therapeutic interchangeability. In the absence of demonstrated differences, practical considerations such as drug availability and cost may reasonably inform the choice between the two agents. Confirmation through prospective, multicenter studies is needed before broad clinical recommendations can be made.

## Figures and Tables

**Table 1:** Baseline characteristics before and after propensity score matching.

Variable	All Patients (n = 154)	Before Matching			After Matching		
		Micafungin (n = 94)	Anidulafungin (n = 60)	*p*-Value	Micafungin (n = 55)	Anidulafungin (n = 55)	*p*-Value
**Demographic and clinical characteristics**							
Female sex, n (%)	69 (44.8)	44 (46.8)	25 (41.7)	0.531	25 (45.5)	25 (45.5)	1.000
Age, years, median (IQR)	70 (61.2–80)	70 (62.2–80.8)	69.5 (59.8–79.2)	0.643	70 (63.5–81.5)	70 (61.5–80.5)	0.711
BMI, kg/m^2^, median (IQR)	25 (23–29)	24.9 (23–28.8)	25 (23–29)	0.840	25.4 (24–29.1)	25 (23–29)	0.374
ICU admission on the day of culture, n (%)	131 (85.1)	81 (86.2)	50 (83.3)	0.630	48 (87.3)	48 (87.3)	1.000
SOFA score, median (IQR)	6 (4–9)	6.5 (4–9)	6 (4–9)	0.910	7 (4–9)	6 (4–9)	0.787
Antifungal use within the preceding month, n (%)	19 (12.3)	12 (12.8)	7 (11.7)	0.840	5 (9.1)	6 (10.9)	0.751
Amphotericin B resistance, n (%)	125 (81.2)	76 (80.9)	49 (81.7)	0.900	46 (83.6)	45 (81.8)	0.801
Fluconazole resistance, n (%)	147 (95.5)	91 (96.8)	56 (93.3)	0.266	54 (98.2)	51 (92.7)	0.182
Concurrent bacteremia, n (%)	17 (11.0)	12 (12.8)	5 (8.3)	0.392	5 (9.1)	5 (9.1)	1.000
Number of antibiotic classes used within the preceding month, median (IQR)	3 (2–4)	3 (2–4)	3 (1–4)	0.244	3 (2–4)	3 (1–4)	0.331
Prior *C. auris* colonization, n (%)	21 (13.6)	16 (17.0)	5 (8.3)	0.126	5 (9.1)	5 (9.1)	1.000
Length of hospital stay before candidemia, days, median (IQR)	25 (14.2–46)	25 (15.2–45.8)	25 (13.5–46.2)	0.907	24 (15–45.5)	24 (13–43.5)	0.962
Time from culture to antifungal initiation, days, median (IQR)	3 (2–4)	3 (2–4)	3 (2–4)	0.885	3 (2–4)	3 (2–4)	0.789
Total duration of antifungal therapy, days, median (IQR)	16 (14–21)	16 (14–21)	16 (14–20)	0.663	16 (14–20)	16 (14–19)	0.685
Length of hospital stay after candidemia, days, median (IQR)	28 (18–54.8)	28 (18–54.5)	27 (17.2–55)	0.936	27 (20–57)	28 (18–48)	0.662
**Comorbidities, n (%)**							
Diabetes mellitus	44 (28.6)	28 (29.8)	16 (26.7)	0.676	17 (30.9)	15 (27.3)	0.675
Arterial hypertension	31 (20.1)	18 (19.1)	13 (21.7)	0.704	8 (14.5)	11 (20.0)	0.449
Coronary artery disease	63 (40.9)	35 (37.2)	28 (46.7)	0.246	24 (43.6)	27 (49.1)	0.566
Chronic liver disease	2 (1.3)	1 (1.1)	1 (1.7)	0.958	1 (1.8)	1 (1.8)	0.752
Chronic kidney disease	14 (9.1)	7 (7.4)	7 (11.7)	0.374	3 (5.5)	6 (10.9)	0.244
COPD	31 (20.1)	18 (19.1)	13 (21.7)	0.704	8 (14.5)	13 (23.6)	0.225
Chronic neurological disease	73 (47.4)	43 (45.7)	30 (50.0)	0.606	28 (50.9)	27 (49.1)	0.849
Acute neurological event	17 (11.0)	11 (11.7)	6 (10.0)	0.742	7 (12.7)	5 (9.1)	0.541
Solid organ malignancy	25 (16.2)	16 (17.0)	9 (15.0)	0.740	11 (20.0)	9 (16.4)	0.621
Hematological malignancy	1 (0.6)	0 (0.0)	1 (1.7)	0.390	0 (0.0)	1 (1.8)	0.500
Immunosuppression (organ transplantation, monoclonal antibody, chemotherapy)	8 (5.2)	4 (4.3)	4 (6.7)	0.380	2 (3.6)	4 (7.3)	0.339
High-dose corticosteroid use	5 (3.2)	1 (1.1)	4 (6.7)	0.076	0 (0.0)	3 (5.5)	0.122
Charlson Comorbidity Index, median (IQR)	5 (4–6)	5 (3.2–6)	5 (4–7)	0.641	5 (4–6.5)	5 (4–7)	0.913
EQUAL *Candida* score, median (IQR) *							
Central venous catheter presence	12 (12–14)	12 (10–15)	12 (10–13)	0.055	12 (10–14)	12 (12–13)	0.276
Central venous catheter absence	10 (10–12)	10 (9–12)	11 (10–12)	0.448	10 (9–12)	11 (10–12)	0.522

Abbreviations: BMI, body mass index; CCI, Charlson Comorbidity Index; IQR, interquartile range; COPD, chronic obstructive pulmonary disease; SOFA, Sequential Organ Failure Assessment; ICU, intensive care unit. * EQUAL *Candida* scores are presented separately for patients with and without a central venous catheter because the maximum achievable score differs between these groups [17].

**Table 2:** Invasive device use and supportive therapies before and after propensity score matching.

Variable, n (%)	All Patients (n = 154)	Before Matching			After Matching		
		Micafungin (n = 94)	Anidulafungin (n = 60)	*p*-Value	Micafungin (n = 55)	Anidulafungin (n = 55)	*p*-Value
**Central venous catheter**	122 (79.2)	75 (79.8)	47 (78.3)	0.828	44 (80.0)	43 (78.2)	0.815
Central venous catheter status *							
Removed	73 (47.4)	45 (47.9)	28 (46.7)	0.976	27 (49.1)	26 (47.3)	0.969
Not removed	49 (31.8)	30 (31.9)	19 (31.7)		17 (30.9)	17 (30.9)	
**Mechanical ventilation**	92 (59.7)	56 (59.6)	36 (60.0)	0.958	33 (60.0)	35 (63.6)	0.695
**Tracheostomy**	43 (27.9)	27 (28.7)	16 (26.7)	0.781	14 (25.5)	14 (25.5)	1.000
**Urinary catheter**	144 (93.5)	90 (95.7)	54 (90.0)	0.142	53 (96.4)	50 (90.9)	0.219
**Surgical drain catheter**	21 (13.6)	15 (16.0)	6 (10.0)	0.293	7 (12.7)	6 (10.9)	0.768
**Vasopressor use**	56 (36.4)	32 (34.0)	24 (40.0)	0.454	21 (38.2)	22 (40.0)	0.845
**Total parenteral nutrition use**	18 (11.7)	14 (14.9)	4 (6.7)	0.121	6 (10.9)	4 (7.3)	0.507
**Abdominal surgery within the preceding 3 months**	9 (5.8)	8 (8.5)	1 (1.7)	0.053	1 (1.8)	1 (1.8)	0.752
**Hemodialysis**	9 (5.8)	6 (6.4)	3 (5.0)	0.508	4 (7.3)	2 (3.6)	0.339

* Catheter removal applies only to patients with a central venous catheter; percentages are calculated based on the total number of patients in the respective column rather than the number of catheterized patients.

**Table 3:** Laboratory parameters on the day of culture before and after propensity score matching.

Variable	All Patients (n = 154)	Before Matching			After Matching		
		Micafungin (n = 94)	Anidulafungin (n = 60)	*p*-Value	Micafungin (n = 55)	Anidulafungin (n = 55)	*p*-Value
WBC, ×10^3^/µL, median (IQR)	10.26 (7.1–13.03)	9.34 (6.81–13.00)	10.60 (7.51–13.07)	0.285	8.82 (7.02–12.28)	10.44 (7.47–12.93)	0.269
Neutrophil count, ×10^3^/µL, median (IQR)	7.42 (5.01–11.14)	7.26 (4.47–10.87)	8.54 (5.69–11.21)	0.189	6.67 (4.67–10.41)	7.75 (5.69–11.10)	0.164
Platelet count, ×10^3^/µL, median (IQR)	240 (161–329)	248 (161–327)	222 (161–334)	0.751	251 (161–344)	211 (146–322)	0.464
Procalcitonin, ng/mL, median (IQR)	0.5 (0.2–1.7)	0.5 (0.2–1.5)	0.6 (0.2–2.0)	0.439	0.5 (0.2–1.3)	0.6 (0.3–2.0)	0.242
CRP, mg/L, median (IQR)	122 (61–190)	126 (66–181)	120 (60–192)	0.997	117 (59–179)	129 (63–194)	0.546
ALT, U/L, median (IQR)	20 (11–35)	19 (11–31)	22 (12–42)	0.508	17 (11–29)	22 (12–42)	0.234

Abbreviations: ALT, alanine aminotransferase; CRP, C-reactive protein; IQR, interquartile range; WBC, white blood cell count.

**Table 4:** Clinical, microbiological, and hepatic safety outcomes in the propensity score–matched cohort.

Outcome	Micafungin (n = 55)	Anidulafungin (n = 55)	Absolute Difference, %	mOR (95% CI)	*p*-Value
**30-day all-cause mortality, n(%)**	23 (41.8)	23 (41.8)	0	1.00 (0.43–2.31)	1.000
**14-day all-cause mortality, n (%)**	9 (16.4)	10 (18.2)	−1.8	0.83 (0.25–2.73)	0.763
**Microbiological response, n (%)**	52 (94.5)	50 (90.9)	+3.6	1.67 (0.40–6.97)	0.480
**End-of-therapy response, n (%)**	41 (74.5)	37 (67.3)	+7.2	1.57 (0.61–4.05)	0.346
**Relapse, n (%)**	7 (12.7)	6 (10.9)	+1.8	1.20 (0.37–3.93)	0.763
**RUCAM category**					
Unlikely, n (%)	1 (1.8)	4 (7.3)	−5.5	-	-
Possible, n (%)	4 (7.3)	2 (3.6)	+3.7	-	-

Abbreviations: CI, confidence interval; mOR, matched odds ratio; RUCAM, Roussel Uclaf Causality Assessment Method. For binary clinical outcomes, mORs were calculated from discordant matched pairs, and *p*-values were obtained using the asymptotic McNemar test. RUCAM categories are presented descriptively; category-specific mORs and *p*-values were not estimated because of sparse cell counts.

## Data Availability

The data presented in this study are available on reasonable request from the corresponding author. The data are not publicly available due to privacy and ethical restrictions related to patient information.

## Supplementary Materials

The following supporting information can be downloaded at https://www.mdpi.com/article/10.3390/jof12070537/s1. Supplementary Table S1. Absolute standardized mean differences of selected propensity score covariates. Supplementary Table S2. Timing of central venous catheter (CVC) removal among catheterized patients, before and after propensity score matching. Supplementary Table S3. Distribution of antibiotic treatments on the day of culture (before and after matching). Supplementary Figure S1. Covariate balance before/after matching.
