# Supplementary material for: Clinical Outcomes of Micafungin and Anidulafungin in Candidozyma auris (Formerly Candida auris) Candidemia: A Propensity Score-Matched Retrospective Cohort Study
Source: J Fungi (Basel). 2026 Jul 22;12(7):537. doi: 10.3390/jof12070537 (PMC13412950; doi:10.3390/jof12070537)
Supplement: Supplementary file 1 [file jof-12-00537-s001.zip › jof-4387479-supplementary.pdf]

Supplementary Table S1. Absolute standardized mean differences of selected propensity score covariates.

Variable	SMD (before matching)	SMD (after matching)
Sex	–0.102	0.000
Age	0.083	0.079
ICU admission (day of culture)	0.082	0.000
CCI	–0.082	–0.008
SOFA score	0.013	0.031
Mechanical ventilation	–0.009	–0.074
Vasopressor use	–0.125	–0.038
Total parenteral nutrition use	0.230	0.099
History of abdominal surgery (within the preceding 3 months)	0.244	0.000
Concurrent bacteremia	0.132	0.000
Time from index culture to antifungal initiation	0.082	–0.011
Prior *C. auris* colonization	0.230	0.000
Length of hospital stay before candidemia	–0.125	0.020
CRP at candidemia onset	–0.020	–0.022

Abbreviations: CCI, Charlson Comorbidity Index; CRP, C-reactive protein; ICU, intensive care unit; SMD, standardized mean difference; SOFA, Sequential Organ Failure Assessment.

Supplementary Table S2. Timing of central venous catheter (CVC) removal among catheterized patients, before and after propensity score matching.

**Timing of CVC removal among catheterized patients†**	**All patients with CVC (n=122)**	**Before matching Micafungin (n=75)**	**Before matching Anidulafungin (n=47)**	***p-value***	**After matching Micafungin (n=44)**	**After matching Anidulafungin (n=43)**	***p-value***
Removed within 24 hours, n (%)	24 (19.7)	18 (24.0)	6 (12.8)	0.263	8 (18.2)	5 (11.6)	0.703
Removed between 24 and 72 hours, n (%)	33 (27.0)	20 (26.7)	13 (27.7)		14 (31.8)	13 (30.2)	
Removed after 72 hours, n (%)	16 (13.1)	7 (9.3)	9 (19.1)		5 (11.4)	8 (18.6)	
Not removed, n (%)	49 (40.2)	30 (40.0)	19 (40.4)		17 (38.6)	17 (39.5)	

†Assessed only among patients with a central venous catheter (CVC) (n = 122). Timing was categorized according to the EQUAL *Candida* score as removal within 24 hours, between 24 and 72 hours, and after 72 hours of diagnosis; patients whose catheter was not removed are shown separately. Percentages use the number of catheterized patients in the corresponding column as the denominator. *p*-values were calculated using Pearson's chi-square test across the four categories.

Supplementary Table S3. Distribution of antibiotic treatments on the day of culture (before and after matching).

	**Before matching**			**After matching**		
**Antibiotic use, n (%)**	**Micafungin (n=94)**	**Anidulafungin (n=60)**	***p*–value**	**Micafungin (n=55)**	**Anidulafungin (n=55)**	***p*–value**
Carbapenem	40 (42.6)	24 (40.0)	0.754	25 (45.5)	22 (40.0)	0.563
Glycopeptide	25 (26.6)	19 (31.7)	0.497	16 (29.1)	17 (30.9)	0.835
Colistin/polymyxin	23 (24.5)	19 (31.7)	0.328	13 (23.6)	18 (32.7)	0.289
Ceftazidime-avibactam	4 (4.3)	0 (0.0)	0.135	1 (1.8)	0 (0.0)	0.315
Piperacillin-tazobactam	10 (10.6)	10 (16.7)	0.278	8 (14.5)	9 (16.4)	0.792
Quinolone	3 (3.2)	5 (8.3)	0.152	0 (0.0)	5 (9.1)	0.067
Macrolide	2 (2.1)	0 (0.0)	0.371	2 (3.6)	0 (0.0)	0.248
Third-generation cephalosporin	3 (3.2)	5 (8.3)	0.152	2 (3.6)	5 (9.1)	0.219
Ampicillin-sulbactam	5 (5.3)	4 (6.7)	0.492	4 (7.3)	4 (7.3)	0.642
Aminoglycoside	5 (5.3)	1 (1.7)	0.245	3 (5.5)	1 (1.8)	0.309
Fosfomycin	4 (4.3)	1 (1.7)	0.352	3 (5.5)	0 (0.0)	0.122

Supplementary Figure S1. Covariate balance before/after matching

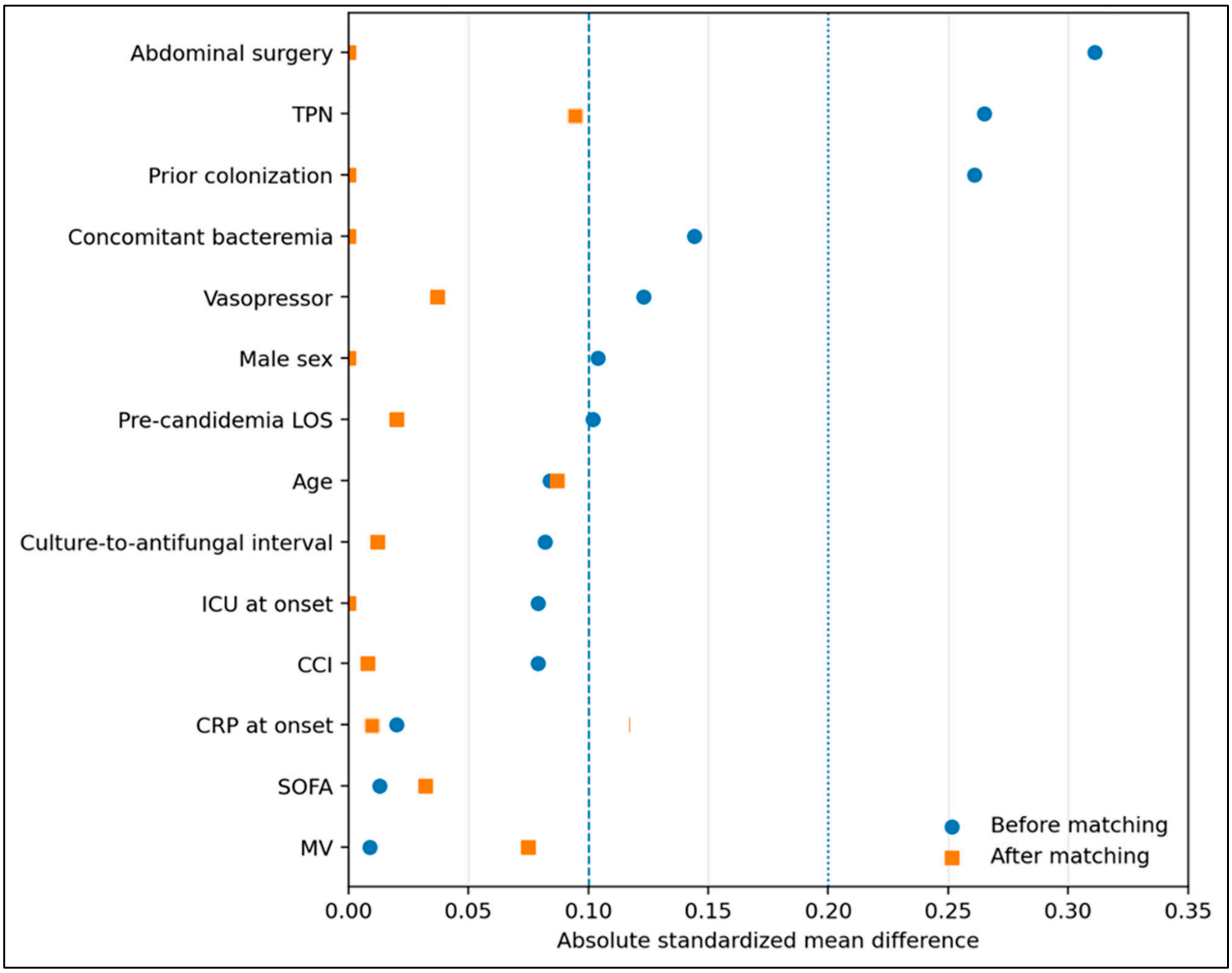

Abbreviations: CCI, Charlson Comorbidity Index; CRP, C-reactive protein; ICU, intensive care unit; LOS, length of stay; MV, mechanical ventilation; SOFA, Sequential Organ Failure Assessment; TPN, total parenteral nutrition.
